# Fibroblasts—a key host cell type in tumor initiation, progression, and metastasis

**DOI:** 10.3109/03009734.2012.654859

**Published:** 2012-04-19

**Authors:** Carina Strell, Helene Rundqvist, Arne Östman

**Affiliations:** Department of Oncology-Pathology, Karolinska Institutet, Stockholm, Sweden

**Keywords:** Cancer associated fibroblasts, cancer stem cell, invasion, metastasis, microenvironment

## Abstract

Tumor initiation, growth, invasion, and metastasis occur as a consequence of a complex interplay between the host environment and cancer cells. Fibroblasts are now recognized as a key host cell type involved in host–cancer signaling. This review discusses some recent studies that highlight the roles of fibroblasts in tumor initiation, early progression, invasion, and metastasis. Some clinical studies describing the prognostic significance of fibroblast-derived markers and signatures are also discussed.

It is now well recognized that tumor initiation, growth, invasion, and metastasis are a consequence of a complex interplay between the host environment and the cancer cell. Among host cells most attention has been given to various immune cells and cells of the vasculature. However, it is now becoming clear that also fibroblasts play crucial roles during various steps in cancer development.

This review discusses some selected and recent studies which highlight and emphasize fibroblasts as receivers and providers of pro-tumoral paracrine signaling. Special attention is given to studies that indicate fibroblasts as critical components of tumor initiation, early progression, and various steps of the metastatic process (Figure 1). Some of these topics have also been covered in other recent reviews (1–6).

**Figure 1.**
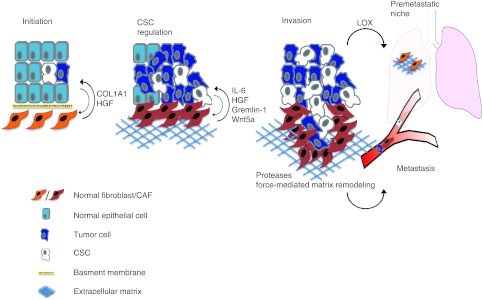
CAF-derived factors can modulate tumor development and progression. The figure schematically illustrates various aspects of tumor initiation progression and metastasis where fibroblasts have been indicated as key regulators. Fibroblasts play a role in tumor initiation and the transition of ductal carcinoma *in situ* towards invasive carcinoma. During tumor progression, CAF-released growth factors and cytokines promote the proliferation of stem cell-like/mesenchymal cells. By the release of proteases and force-mediated remodeling of the extracellular matrix CAFs also support the migration of tumor cells. Activation of fibroblasts is an important component in the formation of pre-metastatic niches.

## Fibroblasts promote early tumor-stimulatory inflammation and ductal carcinoma *in situ* (DCIS) progression

Genetically engineered mouse models of cancer present excellent opportunities to analyze the step-wise progression of cancer development. Like in human cancer, inflammation is observed in the early stages of tumorigenesis in some of these models. An important role of fibroblasts in driving and stimulating this early pro-tumorigenic inflammation was recently uncovered in an analysis using an HPV16-driven model of squamous skin cancer (7). In that study fibroblasts from dysplastic early skin lesions were isolated and found to be characterized by a pro-inflammatory gene signature, including expression of a set of chemokines and interleukins. This signature was also found in cancer-associated fibroblasts (CAFs) from genetic mouse models of breast and pancreatic cancer, and in human CAF preparations. NFkappaB activation was identified as an important factor for maintaining this pro-inflammatory fibroblast phenotype. The exact mechanism(s) inducing the pro-inflammatory fibroblast remain to be characterized, but co-culture experiments with fibroblast and cancer cells indicated that the pro-inflammatory signature in fibroblasts was induced by paracrine signals from cancer cells.

Another highly innovative and original study also identified fibroblasts as an important component in the early stages of cancer (8). This study used as a starting-point epidemiological studies which have established that breast cancers developing close after child-birth are associated with worse prognosis. Since prognosis for these cases is worse than in breast cancer detected during pregnancy it has been suggested that a process subsequent to pregnancy is involved.

The authors hypothesized that the host microenvironment of involuting breast tissue created a progression-permissive microenvironment. The study therefore compared tumor formation, in post-partum mice and nulliparous mice, of low-malignancy breast cancer cells injected into the mammary gland (8). Interestingly, tumor growth was accelerated in post-partum mice, and tumors in this group were also characterized by larger collagen content and a more invasive phenotype. Tissue culture experiments demonstrated that collagen promoted a more aggressive phenotype of the low-malignancy MCF10DCIS cells used in the animal study. The *in-vitro* phenotype induced by collagen was sensitive to cyclo-oxygenase 2 (COX2) blockade, and the *in-vivo* phenotype could also be partially blocked by treatment with COX2 inhibitors. Clinical relevance of these observations was suggested by analyses of gene expression data from breast cancers of younger women, which revealed an association between bad prognosis and high expression of both collagen 1A1 (COL1A1) and COX2 (8). Together these findings suggest important role(s) of fibroblasts, as the major source of collagen production, in the creation of a tumor-permissive host tissue in post-partum breast.

A role for fibroblasts/CAFs in the transition of ductal carcinoma *in situ* to invasive carcinoma was also suggested by another study using co-injection of activated fibroblasts and MCF10DCIS cells. Co-injection of MCF10DCIS cells with either normal human fibroblasts, invasive breast CAFs, or fibroblasts from rheumatoid arthritis resulted in invasive carcinomas, whereas co-injection with myoepithelial cells resulted in ductal carcinoma *in situ* (DCIS) (9). Interestingly, triple injections of MCF10DCIS with myoepithelial cells and fibroblasts/CAFs formed small tumors with DCIS histology, indicating that myoepithelial cells exert a tumor-suppressive effect which cannot be overcome by fibroblasts. It was confirmed that the invasive tumors, derived from fibroblast co-injections, are not formed by expansion of a pre-existing subpopulation of invasive MCF10DCIS cells, since reinjection of tumor cells resulted in DCIS. The results rather indicate that the invasive phenotype is dependent on paracrine fibroblast-mediated signaling.

A third study, linking fibroblast-derived signaling to progression of DCIS, used normal mammary fibroblasts engineered to secrete hepatocyte growth factor (HGF), which was shown to increase the invasiveness of MCF10DCIS cells (10). To mimic *in-vivo* DCIS outgrowth and progression the group used a three-dimensional cell culture method originally established in the Bissell and Brugge laboratories (11,12). MCF10DCIS cultures including HGF-expressing fibroblasts, or conditioned medium from these fibroblasts, showed a more invasive growth pattern than cultures without, or with control fibroblasts. *In-vivo* relevance of these findings was confirmed by co-injection xenograft experiments. The mechanisms of HGF-induced aggressiveness were shown to include an increased expression and secretion of urokinase-type plasminogen activator (uPa) and uPAR (13,14), increased collagen IV degradation, and an increased migratory phenotype in the MCF10DCIS.

Together these three studies on DCIS suggest that fibroblasts exert important stimulatory roles in the progression of DCIS. Future studies are likely to investigate if fibroblasts, in addition to having direct effects on the DCIS cells, also play an active part in the disruption of the postulated tumor-suppressive effects of myoepithelial cells.

## Fibroblasts drive tumor progression by modulation of biomechanical forces

Biomechanical factors are known to control tissue development and modulate tissue homeostasis. The extracellular matrix, predominantly derived from fibroblasts, is one key determinant of the biomechanical properties of tumors. Recent studies have demonstrated how modulation of these properties can act as a promoter of tumor progression and have also started to uncover the signaling pathways that are involved in conveying the mechanical alterations into changes of cell phenotypes.

A landmark study by Levental et al. showed that cross-linked matrix and the associated increase in ‘stiffness' might be a key driver of tumor progression (15). A mammary gland microenvironment with increased matrix cross-linking was established by implantation of fibroblasts over-expressing lysyl oxidase (LOX). Following injection of H-ras expressing premalignant cancer cells, there was an increased tumor growth and invasion in mice with cross-linked matrix. Furthermore, treatment with a LOX inhibitor reduced tumor growth in the MMTV-neu breast cancer model. Subsequent tissue culture studies using untransformed mammary epithelial cells revealed that culture on cross-linked matrix was associated with disruption of well-organized gland-like structures. Also, cells with activated ErbB2 formed invasive structures only on chemically cross-linked matrix. Together these findings indicate modulation of extracellular matrix stiffness as a potential mechanism whereby fibroblasts can regulate tumor initiation and progression.

This notion recently received additional support through careful analyses of fibroblasts from caveolin (cav) knock-out mice (16). It was demonstrated that cav^-/-^ fibroblasts formed an extracellular matrix characterized by reduced stiffness and reduced fibronectin fiber parallelism. Culture of cancer cells in extracellular matrix (ECM) from wild-type (wt) or cav^-/-^ fibroblast demonstrated that the cav^-/-^ ECM was less permissive for tumor cell migration and invasion. Studies were expanded to *in-vivo* experiments, which showed reduced tumor invasion when cancer cells were implanted in cav^-/-^ animals. Furthermore, co-injection studies showed that tumors derived from cancer cells co-injected with cav^-/-^ fibroblasts displayed reduced ability to form metastases. Associations between reduced caveolin expression and bad prognosis were also demonstrated, in agreement with previous larger studies on stromal caveolin and breast cancer prognosis (17).

## CAF-dependent support of cancer stem cells (CSCs)

Emerging evidence for a strong niche dependency of normal stem cells has prompted studies on the roles of mesenchymal cells in the regulation of cancer stem cells. Such studies are also motivated by recent demonstrations that soluble factors, such as Wnt, Notch, bone morphogenetic proteins (BMPs), hedgehog and various cytokines, support cancer stem cells (CSCs) (18,19). Further, CAF-stimulatory effects on CSCs are also suggested by the recent demonstration that CSCs can obtain an ‘autonomous' state by exposure to certain combinations of growth factors (20). Finally, previous analyses of clinical samples have demonstrated that the CSC marker nuclear beta-catenin is predominantly observed in tumor cells at the invasive margins where CAFs are located at high density and in close proximity to tumor cells (21).

In accordance with these ideas Vermeulen et al. recently described a colon cancer stem cell niche involving HGF released by colon CAFs, which induced a beta-catenin-dependent gene transcription and CSC clonogenicity (22). This study used primary human CAFs isolated from different primary resected human colon carcinomas and also a colonic myofibroblast cell line. Colon CSCs were identified using a TCF/LEF reporter directing Wnt-dependent expression of EGFP. High reporter gene activity was also associated with high expression of colon CSC markers like CD133+, CD166+/CD44+, and up-regulation of the c-Met receptor. Importantly, CAF-induced CSCs showed higher clonogenicity and increased tumor-forming capacity in subcutaneous xenograft mouse models. HGF was implied as a key mediator of this process since the Wnt-reporter-stimulatory activity of myofibroblast conditioned medium was blocked by neutralizing HGF antibodies. This is in agreement with previous studies which have shown that HGF is a potent inducer of epithelial to mesenchymal transition (EMT) (23,24) and recent studies highlighting the overlap of EMT and CSC phenotypes (20). Myofibroblasts secreting HGF could also induce a CSC phenotype in differentiated tumor cells, indicating a bidirectional path between CSCs and more differentiated tumor cells. This finding agrees with other recent data showing that a subpopulation of basal-like human mammary epithelial cells can spontaneously dedifferentiate into stem-like cells (25).

An important effect of CAFs on CSCs was also emphasized in a recent study describing bone-marrow-derived CAFs in gastric cancer (26). This study used as a starting-point clinical findings demonstrating that CAFs of gastric cancer are, at least partially, of bone-marrow origin (27). Using different mouse models for inflammation-induced gastric cancer, the study of Quante et al. showed that CAF precursors appeared in the bone-marrow already during chronic inflammation and increased during carcinogenesis. These smooth muscle actin (ASMA)-positive cells, possibly derived from mesenchymal stem cells in a TGFβ-dependent manner, were more potent than non-bone-marrow-derived fibroblasts in promotion of tumor growth and dissemination (26). An important effect of these cells on CSCs was inferred by their high secretion of stemness-factors such as Wnt5a, gremlin-1, and IL-6 (18,20,28,29).

Studies from the Kuperwasser group have also recently demonstrated the ability of fibroblasts to enhance tumorigenicity and stemcellness of cancer cells (30). Using primary fibroblasts isolated from human breast tumors or from normal breast tissue, it was shown that fibroblasts, which have a high prostaglandin E2 (PGE2) secretion *in vitro*, exhibit a strong tumor-promoting ability *in vivo* when orthotopically co-injected with MCF7 cells into SCID mice. The PGE2 expression seemed to be related to the stem cell-inducing capacity of fibroblasts through autocrine mechanisms, including induction of IL-6 in fibroblasts, which would then act on cancer cells and promote the expansion of cells expressing a breast CSC signature (EpCAM+/CD24-/CD44+). An interesting and somewhat surprising finding in this study was that PEG2 expression and the ability to induce CSCs were not strongly associated with the origin of the fibroblasts.

Taken together, these studies indicate CAFs as important components of a cancer stem cell niche providing regulating factors such as IL-6, BMP antagonists, and factors activating canonical and non-canonical Wnt signaling. Based on the strong evidence for a niche-dependent regulation of CSCs, it is justified to suggest targeting of these interactions as a strategy for therapeutic CSC depletion.

## Involvement of fibroblasts in invasion

Elegant *in-vitro* studies from the Sahai laboratory have suggested that fibroblasts act as important components in cancer tissue invasion. Advanced imaging of cancer cell *in-vitro* invasion, using co-cultures of fibroblasts and cancer cells, demonstrated that fibroblasts acted as leading cells during invasion (31). Formation of invasion-permissive tracks was concluded to be a major mechanism whereby fibroblasts in this model system promoted invasion. These tracks were formed by a combination of protease and force-mediated matrix remodeling. In the original study integrin alpha and Rho-mediated regulation of myosin light chain activity were identified as critical components of the pro-invasive effects of fibroblasts. More recent studies have further explored the cell biology underlying this phenomenon and also explored the possibility to target this process with chemical libraries.

A chemical screen designed to identify agents that blocked fibroblast-mediated matrix remodeling demonstrated activity of lovastatin and simstatin. Their activity was shown to be related to the ability to interfere with the function of Rab proteins (32). Subsequent analyses identified Rab21, through its impact on integrin alpha5 cell surface localization, as a particularly important factor for the ability of fibroblasts to support and stimulate invasion of squamous cell carcinoma. Using a siRNAscreen approach, LIM kinases were also shown to be critical for the ability of fibroblasts to lead invasion. Recently, cytokine dependent track formation of fibroblasts was suggested. Implied pathways ultimately affecting actomyosin contractility, include the receptor gp130-IL6ST, JAK1, and rho-kinases (33).

## Involvement of fibroblasts in the pre-metastatic niche and as targets for instigating signaling

**Figure 2.**
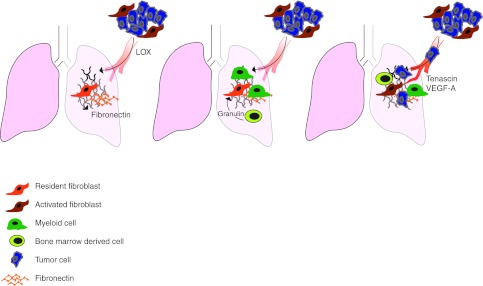
Fibroblast involvement in the formation of a metastasis-permissive microenvironment. A: Local fibroblasts respond to systemic signals by depositing fibronectin in the target organ and thereby priming it for homing of myeloid cells and subsequent metastatic settlement. In an alternative pathway of metastatic initiation, LOX is secreted from the primary tumor to cross-link collagen IV in fibronectin-rich areas of the target organ. B: The ‘primed' microenvironment of the pre-metastatic site promotes recruitment of myeloid cells and subsequently also bone-marrow-derived cells and tumor cells. C: In response to systemic signaling, mobilized bone-marrow cells are recruited to the secondary site. Through a granulin-dependent mechanism they can then activate local fibroblasts. Presence of activated fibroblasts, producing VEGF-A and tenascin, supports angiogenesis in metastasis growth.

Recent studies have suggested that activation of fibroblasts is a key event in formation of the pre-metastatic niche. The concept of such a niche was introduced in 2005 by Lyden and colleagues following findings that systemic signals, involving humoral factors, from the primary tumor prepare a niche in target organs that become primed for subsequent settlement and growth of metastasis (34). A number of steps in this process were identified, including an increased deposition of fibroblast-derived fibronectin in the host microenvironment (Figure 2). Interestingly, mouse lung carcinoma (LLC) and B16 melanoma initiated such clusters in different organs in a manner which paralleled their respective metastatic patterns. Also, pre-injection of mice with melanoma-conditioned media redirected LLC metastasis, following tail-vein injection of such cells, to a more melanoma-like pattern of spreading.

One of the tumor-secreted factors suggested to initiate the metastatic niche is LOX. The amine oxidase LOX was identified when screening for genes associated with hypoxia-induced metastasis (35). LOX was previously known to cross-link collagens and elastins in the ECM but had also been associated with increased breast cancer cell invasion *in vitro* (36). In a mouse model of breast cancer, inhibition of LOX reduced cancer cell motility and invasiveness and prevented metastasis (37). High expression of LOX correlates with poor prognosis in ER-negative breast cancer patients and in patients with head and neck cancer (37). The pro-metastatic effect of LOX, in the pre-metastatic niche, appears to be secondary to the activation of fibroblasts, since LOX deposition and enzymatic activity are concentrated to areas of fibronectin deposits (38,39). The niche-promoting effects of LOX include cross-linking of collagen IV that recruits first myeloid cells and then bone-marrow-derived cells and tumor cells (39). A more recent study has provided further support for a link between the fibroblast-initiated pre-metastatic niche and the therapeutic effects of LOX inhibitors, by showing that LOX inhibitors have no effect on established metastasis (40).

Another member of the lysyl oxidase family, LOXL2, also shows tumor-promoting effects. Like LOX it can cross-link fibrillar collagen and contribute to invasion (41). An experimental study recently investigated the effects of a LOXL2-specific monoclonal antibody on tumor characteristics (42). The antibody showed inhibitory effects on LOXL2-induced EMT-like remodeling of MCF7 cells and fibroblast-induced tube formation in HUVECs. It also inhibited tumor growth in an orthotopic breast cancer model. Histological analyses of tumors showed less cross-linked collagen matrix, fewer ASMA-positive fibroblasts, and reduced microvessel density. Analyses of growth factor production showed lower amounts of VEGF-A, CXCL12, and TGFβ1 in tumors of mice treated with the monoclonal antibody. Treatment also reduced metastasis formation in two different mouse models of metastasis (42).

The group of McAllister recently described another mechanism of systemic activation of the local stroma and subsequent progression of an idle tumor cell population (43). Using the instigator–responder model, they showed that the instigating effects of activated bone-marrow cells include activation and stimulation of fibroblasts (Figure 2). Granulin, acting on fibroblasts, was identified as a key mediator in the instigating activity of the activated bone-marrow cells. Expression analyses of the activated fibroblasts revealed a pro-inflammatory profile. The authors thus concluded that the instigating effect of the granulin-expressing bone-marrow-derived cells involves activation of fibroblasts at distant sites (43).

Mice engineered to express a suicide gene in a certain cell type allow studies where the importance of a particular cell type in a given process can be investigated. The Kalluri group has used this strategy and made a panel of mice where thymidine-kinase (TK) is expressed under various promoters, including S100A4, that are active in different fibroblast subsets. This group recently used the orthotopic 4T1 breast cancer model to show that depletion of S100A4 cells reduced the metastatic area, increased apoptosis in metastases, and reduced CD31 staining (44). A similar dependence of S100A4-positive cells was identified in experiments with tail-vein injections of 4T1 cells and in a metastatic colorectal cancer model.

Analyses of control breast metastases in the lung revealed that S100A4-positive cells normally are present in low numbers in lung tissue but accumulate after orthotopic injection. Some of these S100A4-positive cells were CD45 + bone-marrow cells, but a majority was considered to be activated resident fibroblasts. This fibroblast population was shown to express fibronectin, tenascin-C, and VEGF-A. S100A4-positive cells, which did not express the CD45 immune cell marker, were also detected in the metastatic microenvironment of human breast cancers (44).

## Clinical correlations

An obvious implication of the studies discussed above is that cancer prognosis is determined by characteristics of cancer-associated fibroblasts. This notion is presently being supported by a large number of studies that have explored the prognostic ability of various CAF proteins or signatures. A few studies are discussed below to exemplify different approaches that have been used to identify fibroblast-derived prognostic factors.

Platelet-derived growth factor (PDGF) receptors are well-established regulators of CAFs. Innovative and early studies from the Westermark group provided the first evidence that activation of stromal PDGF receptors could support tumor growth (45). Studies in animal models have demonstrated that targeting of PDGF receptors in CAFs has major effects on tumor growth and drug delivery (46–48). Analyses of larger series of clinical material have now also implied PDGF receptor signaling in metastasis, since significant associations were detected between stromal PDGF beta-receptor expression and prognosis in breast and prostate cancer (49,50).

The last-mentioned analyses, which relied on immunohistochemistry, have now received further support through a gene expression-based study. In this study a gene signature, reflecting activation of stromal PDGF receptors, was used as a classifier of high score predicted survival? Unclear whether good or poor survival multiple large breast cancer gene expression data sets (Frings et al., in preparation). Interestingly, a high PDGFR signature score emerged as a significant predictor of breast cancer survival in multivariate analyses, including standard clinical characteristics such as hormone receptor status, grade, and tumor size.

An alternative stroma gene expression signature was published in 2009 that predicted prognosis in breast cancer. This signature was established by comparing gene expression in the stroma of bad and good prognosis cases of breast cancers (51). A related, but distinct, approach was recently used to generate a prognostic fibroblast-related signature in lung cancer. In this case gene expression was compared in a panel of 15 matched pairs of normal lung fibroblasts and non-small cell lung cancer (NSCLC) CAFs, and a number of consistently up-regulated genes were identified (52). A classifier was subsequently made, composed of a subset of these genes, that demonstrated prognostic capacity in multiple NSCLC data sets. In this case the underlying biology remains to be fully characterized, but it was noted that many of the genes of the classifier were up-regulated upon TGFβ stimulation of fibroblasts. In another lung cancer study stromal expression of the transcription factor FoxF1, shown to act as a potent inducer of a CAF-like phenotype of fibroblasts, was found to separate patients with large cell lung cancer into groups with different prognosis (53).

## Concluding outlook

Fibroblasts have found a solid position as a cell type to be considered in analyses of cancer cell–host interactions. However, a series of challenges remain. In general, an improved classification of functionally relevant fibroblast subsets remains a key issue. Also, it is highly motivated to establish standardized model systems where *in-situ* characteristics of such functional subsets are preserved and possible to monitor.

A number of emerging areas can be identified, including studies of circulating fibroblasts and their role in metastasis (54,55), the roles of chemokines in regulation of fibroblasts (56), and possible metabolic interactions between CAFs and e.g. hypoxic cancer cells (57,58). Recent experimental and clinical studies also indicate fibroblasts as key regulators of cancer cell drug sensitivity (59,60). Most likely this field of research will expand.

In the future it will also be important to analyze interactions between host fibroblasts and early tumor-initiating cells. Such interactions are likely to involve both tumor-inhibitory and -promoting effects (61). Enhancement of potential fibroblast-derived tumor-inhibitory effects appears as a highly attractive and yet unexplored strategy for chemoprevention.

During preparation of this manuscript additional support for important CAF/cancer stem cell cross-talk has been provided in articles by Lonardo et al., Cancer Cell, 2011 (DOI 10.1016) and Malanchi et al, Nature, 2011 (DOI 10.1038).

## References

[1] Ostman A, Augsten M (2009). Cancer-associated fibroblasts and tumor growth—bystanders turning into key players. Curr Opin Genet Dev.

[2] Pietras K, Ostman A (2010). Hallmarks of cancer: interactions with the tumor stroma. Exp Cell Res.

[3] McAllister SS, Weinberg RA (2010). Tumor-host interactions: a far-reaching relationship. J Clin Oncol.

[4] Bergfeld SA, DeClerck YA (2010). Bone marrow-derived mesenchymal stem cells and the tumor microenvironment. Cancer Metastasis Rev.

[5] Korkaya H, Liu S, Wicha MS (2011). Breast cancer stem cells, cytokine networks, and the tumor microenvironment. J Clin Invest.

[6] Calvo F, Sahai E (2011). Cell communication networks in cancer invasion. Curr Opin Cell Biol.

[7] Erez N, Truitt M, Olson P, Arron ST, Hanahan D (2010). Cancer-associated fibroblasts are activated in incipient neoplasia to orchestrate tumor-promoting inflammation in an NF-kappaB-dependent manner. Cancer Cell.

[8] Lyons TR, O'Brien J, Borges VF, Conklin MW, Keely PJ, Eliceiri KW (2011). Postpartum mammary gland involution drives progression of ductal carcinoma in situ through collagen and COX-2. Nat Med.

[9] Hu M, Yao J, Carroll DK, Weremowicz S, Chen H, Carrasco D (2008). Regulation of in situ to invasive breast carcinoma transition. Cancer Cell.

[10] Jedeszko C, Victor BC, Podgorski I, Sloane BF (2009). Fibroblast hepatocyte growth factor promotes invasion of human mammary ductal carcinoma in situ. Cancer Res.

[11] Debnath J, Brugge JS (2005). Modelling glandular epithelial cancers in three-dimensional cultures. Nat Rev Cancer.

[12] Lee GY, Kenny PA, Lee EH, Bissell MJ (2007). Three-dimensional culture models of normal and malignant breast epithelial cells. Nat Methods.

[13] Jeffers M, Rong S, Vande Woude GF (1996). Enhanced tumorigenicity and invasion-metastasis by hepatocyte growth factor/scatter factor-met signalling in human cells concomitant with induction of the urokinase proteolysis network. Mol Cell Biol.

[14] Pepper MS, Matsumoto K, Nakamura T, Orci L, Montesano R (1992). Hepatocyte growth factor increases urokinase-type plasminogen activator (u-PA) and u-PA receptor expression in Madin-Darby canine kidney epithelial cells. J Biol Chem.

[15] Levental KR, Yu H, Kass L, Lakins JN, Egeblad M, Erler JT (2009). Matrix crosslinking forces tumor progression by enhancing integrin signaling. Cell.

[16] Goetz JG, Minguet S, Navarro-Lerida I, Lazcano JJ, Samaniego R, Calvo E (2011). Biomechanical remodeling of the microenvironment by stromal caveolin-1 favors tumor invasion and metastasis. Cell.

[17] Witkiewicz AK, Dasgupta A, Sotgia F, Mercier I, Pestell RG, Sabel M (2009). An absence of stromal caveolin-1 expression predicts early tumor recurrence and poor clinical outcome in human breast cancers. Am J Pathol.

[18] Liu S, Ginestier C, Ou SJ, Clouthier SG, Patel SH, Monville F (2011). Breast cancer stem cells are regulated by mesenchymal stem cells through cytokine networks. Cancer Res.

[19] Medema JP, Vermeulen L (2011). Microenvironmental regulation of stem cells in intestinal homeostasis and cancer. Nature.

[20] Scheel C, Eaton EN, Li SH, Chaffer CL, Reinhardt F, Kah KJ (2011). Paracrine and autocrine signals induce and maintain mesenchymal and stem cell states in the breast. Cell.

[21] Brabletz T, Jung A, Reu S, Porzner M, Hlubek F, Kunz-Schughart LA (2001). Variable beta-catenin expression in colorectal cancers indicates tumor progression driven by the tumor environment. Proc Natl Acad Sci USA.

[22] Vermeulen L, De Sousa EMF, van der Heijden M, Cameron K, de Jong JH, Borovski T (2010). Wnt activity defines colon cancer stem cells and is regulated by the microenvironment. Nat Cell Biol.

[23] Muller T, Bain G, Wang X, Papkoff J (2002). Regulation of epithelial cell migration and tumor formation by beta-catenin signaling. Exp Cell Res.

[24] Rasola A, Fassetta M, De Bacco F, D'Alessandro L, Gramaglia D, Di Renzo MF (2007). A positive feedback loop between hepatocyte growth factor receptor and beta-catenin sustains colorectal cancer cell invasive growth. Oncogene.

[25] Chaffer CL, Brueckmann I, Scheel C, Kaestli AJ, Wiggins PA, Rodrigues LO (2011). Normal and neoplastic nonstem cells can spontaneously convert to a stem-like state. Proc Natl Acad Sci USA.

[26] Quante M, Tu SP, Tomita H, Gonda T, Wang SS, Takashi S (2011). Bone marrow-derived myofibroblasts contribute to the mesenchymal stem cell niche and promote tumor growth. Cancer Cell.

[27] Worthley DL, Ruszkiewicz A, Davies R, Moore S, Nivison-Smith I, Bik To L (2009). Human gastrointestinal neoplasia-associated myofibroblasts can develop from bone marrow-derived cells following allogeneic stem cell transplantation. Stem Cells.

[28] Sansone P, Storci G, Tavolari S, Guarnieri T, Giovannini C, Taffurelli M (2007). IL-6 triggers malignant features in mammospheres from human ductal breast carcinoma and normal mammary gland. J Clin Invest.

[29] Sneddon JB, Zhen HH, Montgomery K, van de Rijn M, Tward AD, West R (2006). Bone morphogenetic protein antagonist gremlin 1 is widely expressed by cancer-associated stromal cells and can promote tumor cell proliferation. Proc Natl Acad Sci USA.

[30] Rudnick JA, Arendt LM, Klebba I, Hinds JW, Iyer V, Gupta PB (2011). Functional heterogeneity of breast fibroblasts is defined by a prostaglandin secretory phenotype that promotes expansion of cancer-stem like cells. PLoS ONE.

[31] Gaggioli C, Hooper S, Hidalgo-Carcedo C, Grosse R, Marshall JF, Harrington K (2007). Fibroblast-led collective invasion of carcinoma cells with differing roles for RhoGTPases in leading and following cells. Nat Cell Biol.

[32] Scott RW, Hooper S, Crighton D, Li A, Konig I, Munro J (2010). LIM kinases are required for invasive path generation by tumor and tumor-associated stromal cells. J Cell Biol.

[33] Samuel MS, Lopez JI, McGhee EJ, Croft DR, Strachan D, Timpson P (2011). Actomyosin-mediated cellular tension drives increased tissue stiffness and beta-catenin activation to induce epidermal hyperplasia and tumor growth. Cancer Cell.

[34] Kaplan RN, Riba RD, Zacharoulis S, Bramley AH, Vincent L, Costa C (2005). VEGFR1-positive haematopoietic bone marrow progenitors initiate the pre-metastatic niche. Nature.

[35] Denko NC, Fontana LA, Hudson KM, Sutphin PD, Raychaudhuri S, Altman R (2003). Investigating hypoxic tumor physiology through gene expression patterns. Oncogene.

[36] Kirschmann DA, Seftor EA, Fong SF, Nieva DR, Sullivan CM, Edwards EM (2002). A molecular role for lysyl oxidase in breast cancer invasion. Cancer Res.

[37] Erler JT, Bennewith KL, Nicolau M, Dornhofer N, Kong C, Le QT (2006). Lysyl oxidase is essential for hypoxia-induced metastasis. Nature.

[38] Fogelgren B, Polgar N, Szauter KM, Ujfaludi Z, Laczko R, Fong KS (2005). Cellular fibronectin binds to lysyl oxidase with high affinity and is critical for its proteolytic activation. J Biol Chem.

[39] Erler JT, Bennewith KL, Cox TR, Lang G, Bird D, Koong A (2009). Hypoxia-induced lysyl oxidase is a critical mediator of bone marrow cell recruitment to form the premetastatic niche. Cancer Cell.

[40] Bondareva A, Downey CM, Ayres F, Liu W, Boyd SK, Hallgrimsson B (2009). The lysyl oxidase inhibitor, beta-aminopropionitrile, diminishes the metastatic colonization potential of circulating breast cancer cells. PLoS ONE.

[41] Csiszar K (2001). Lysyl oxidases: a novel multifunctional amine oxidase family. Prog Nucleic Acid Res Mol Biol.

[42] Barry-Hamilton V, Spangler R, Marshall D, McCauley S, Rodriguez HM, Oyasu M (2010). Allosteric inhibition of lysyl oxidase-like-2 impedes the development of a pathologic microenvironment. Nat Med.

[43] Elkabets M, Gifford AM, Scheel C, Nilsson B, Reinhardt F, Bray MA (2011). Human tumors instigate granulin-expressing hematopoietic cells that promote malignancy by activating stromal fibroblasts in mice. J Clin Invest.

[44] O'Connell JT, Sugimoto H, Cooke VG, Macdonald BA, Mehta AI, Lebleu VS (2011). VEGF-A and Tenascin-C produced by S100A4+ stromal cells are important for metastatic colonization. Proc Natl Acad Sci USA.

[45] Forsberg K, Valyi-Nagy I, Heldin CH, Herlyn M, Westermark B (1993). Platelet-derived growth factor (PDGF) in oncogenesis: development of a vascular connective tissue stroma in xenotransplanted human melanoma producing PDGF-BB. Proc Natl Acad Sci USA.

[46] Pietras K, Ostman A, Sjoquist M, Buchdunger E, Reed RK, Heldin CH (2001). Inhibition of platelet-derived growth factor receptors reduces interstitial hypertension and increases transcapillary transport in tumors. Cancer Res.

[47] Pietras K, Rubin K, Sjoblom T, Buchdunger E, Sjoquist M, Heldin CH (2002). Inhibition of PDGF receptor signaling in tumor stroma enhances antitumor effect of chemotherapy. Cancer Res.

[48] Pietras K, Hanahan D (2005). A multitargeted, metronomic, and maximum-tolerated dose "chemo-switch" regimen is antiangiogenic, producing objective responses and survival benefit in a mouse model of cancer. J Clin Oncol.

[49] Hagglof C, Hammarsten P, Josefsson A, Stattin P, Paulsson J, Bergh A (2010). Stromal PDGFRbeta expression in prostate tumors and non-malignant prostate tissue predicts prostate cancer survival. PLoS ONE.

[50] Paulsson J, Sjoblom T, Micke P, Ponten F, Landberg G, Heldin CH (2009). Prognostic significance of stromal platelet-derived growth factor beta-receptor expression in human breast cancer. Am J Pathol.

[51] Wennmalm K, Ostman A, Bergh J (2009). Stromal signature identifies basal breast cancers. Nat Med.

[52] Navab R, Strumpf D, Bandarchi B, Zhu CQ, Pintilie M, Ramnarine VR (2011). Prognostic gene-expression signature of carcinoma-associated fibroblasts in non-small cell lung cancer. Proc Natl Acad Sci USA.

[53] Saito RA, Micke P, Paulsson J, Augsten M, Pena C, Jonsson P (2010). Forkhead box F1 regulates tumor-promoting properties of cancer-associated fibroblasts in lung cancer. Cancer Res.

[54] Duda DG, Duyverman AM, Kohno M, Snuderl M, Steller EJ, Fukumura D (2010). Malignant cells facilitate lung metastasis by bringing their own soil. Proc Natl Acad Sci USA.

[55] Grum-Schwensen B, Klingelhofer J, Berg CH, El-Naaman C, Grigorian M, Lukanidin E (2005). Suppression of tumor development and metastasis formation in mice lacking the S100A4(mts1) gene. Cancer Res.

[56] Augsten M, Hagglof C, Olsson E, Stolz C, Tsagozis P, Levchenko T (2009). CXCL14 is an autocrine growth factor for fibroblasts and acts as a multi-modal stimulator of prostate tumor growth. Proc Natl Acad Sci USA.

[57] Pavlides S, Whitaker-Menezes D, Castello-Cros R, Flomenberg N, Witkiewicz AK, Frank PG (2009). The reverse Warburg effect: aerobic glycolysis in cancer associated fibroblasts and the tumor stroma. Cell Cycle.

[58] Koukourakis MI, Giatromanolaki A, Harris AL, Sivridis E (2006). Comparison of metabolic pathways between cancer cells and stromal cells in colorectal carcinomas: a metabolic survival role for tumor-associated stroma. Cancer Res.

[59] Farmer P, Bonnefoi H, Anderle P, Cameron D, Wirapati P, Becette V (2009). A stroma-related gene signature predicts resistance to neoadjuvant chemotherapy in breast cancer. Nat Med.

[60] McMillin DW, Delmore J, Weisberg E, Negri JM, Geer DC, Klippel S (2010). Tumor cell-specific bioluminescence platform to identify stroma-induced changes to anticancer drug activity. Nat Med.

[61] Flaberg E, Markasz L, Petranyi G, Stuber G, Dicso F, Alchihabi N (2011). High-throughput live-cell imaging reveals differential inhibition of tumor cell proliferation by human fibroblasts. Int J Cancer.

